# An Uncommon Thyroid Tumor: The Diagnostic Challenge of Solitary Fibrous Tumors

**DOI:** 10.3390/biomedicines14040803

**Published:** 2026-04-01

**Authors:** Rosa Lauretta, Giulia Puliani, Marta Bianchini, Marilda Mormando, Antonietta Fasciglione, Maria Flavia Bagaglini, Ferdinando Marandino, Marialuisa Appetecchia

**Affiliations:** Oncological Endocrinology Unit, IRCCS Regina Elena National Cancer Institute, 00144 Rome, Italy; giulia.puliani@ifo.it (G.P.); marta.bianchini@ifo.it (M.B.); marilda.mormando@ifo.it (M.M.); antonietta.fasciglione@ifo.it (A.F.); mariaflavia.bagaglini@ifo.it (M.F.B.); ferdinando.marandino@ifo.it (F.M.); marialuisa.appetecchia@ifo.it (M.A.)

**Keywords:** thyroid carcinoma, solitary fibrous tumor, NAB2-STAT6 fusion, molecular diagnostics, precision oncology

## Abstract

**Background**: Solitary fibrous tumors are uncommon fibroblastic neoplasms. These tumors are characterized by the recurrent NAB2-STAT6 gene fusion, which is a hallmark of solitary fibrous tumors (SFTs), particularly those arising in the thoracic cavity. While SFTs are mostly found in the abdomen and pleura, they can occur in various locations, including the head and neck region (6% of cases of SFTs). Solitary fibrous tumors of the thyroid (SFTTs) are extremely rare, accounting for only 0.1% of all thyroid tumors. The gold standard imaging modality for thyroid tumors is ultrasonography, even though distinctive characteristics for these types of neoplasms are absent, making pre-operative diagnosis more challenging. **Aim**: The aim of this study is to perform a systematic literature review and to describe our case by analyzing the main clinical features, histological diagnostic features and treatments of this rare tumor, in order to clarify the behavior and molecular characteristics of SFTTs. **Methods**: A comprehensive systematic literature review was conducted according to the PRISMA guidelines for SFTTs. We searched the PubMed and EMBASE databases for articles published up to November 2025. The inclusion criteria include confirmed diagnosis of SFTT, while articles describing unrelated neoplasms or articles that were not in English were excluded. A standardized form was used to extract information on the imaging characteristics, histological diagnosis, treatment and outcome. **Results**: As of 2025, a total of 43 articles were selected, with 61 reported cases of SFTT in the English literature. Pre-operative diagnosis of SFTT is controversial and usually requires immunohistochemical confirmation. In our case, molecular analysis identified, for the first time, a NAB2ex6–STAT6ex17 fusion, contributing to the molecular characterization of this rare tumor. **Conclusions**: SFTTs are rare and difficult to diagnose; thus, they require a multidisciplinary approach for accurate diagnosis and management. The combination of imaging, cytology, histopathology, and molecular testing is essential in distinguishing SFTTs from other thyroid malignancies. Surgical excision remains the mainstay of treatment, and long-term follow-up is recommended due to the potential risk of recurrence or metastasis.

## 1. Introduction

Solitary fibrous tumors (SFTs) are fibroblastic neoplasms that are classified into two categories by the World Health Organization (WHO): those originating in the central nervous system (CNS) and those occurring outside the CNS. These tumors are characterized by the recurrent NAB2-STAT6 gene fusion, which is a hallmark of SFTs, particularly those arising in the thoracic cavity [1,2]. While SFTs are most often found in the pleura, they can occur in various other body parts, including the head and neck region (6% of cases of SFTs) [3]. Among extra CNS, the most frequent localizations are the abdomen (31%), limbs (29%), pleura (22%), trunk (11%) and head and neck (6%) [4]. Solitary fibrous tumors of the thyroid (SFTTs) are exceedingly rare, accounting for only 0.1% of thyroid tumors. Ultrasonography lacks distinct radiological features for this type of neoplasm, further complicating pre-operative diagnosis [5,6,7,8]. Here, we present our case, analyzing the main clinical features, histological diagnostic challenges, and treatments of this rare localization of SFTT, and providing an in-depth and detailed systematic review of the literature.

## 2. Materials and Methods

**Figure 1:**
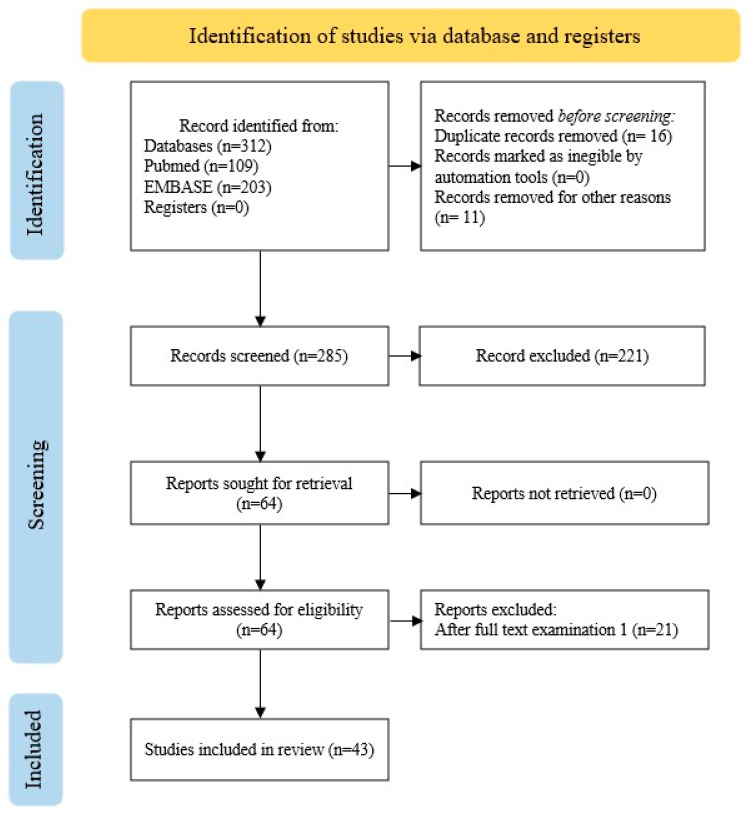
PRISMA flow diagram.

A comprehensive systematic literature review was conducted according to the PRISMA guidelines [9]. We searched PubMed and EMBASE for articles published up to 2025 on SFTTs. The following search terms were used for PubMed: “solitary fibrous tumor*” OR “solitary fibrous tumor*” OR SFT AND thyroid. For EMBASE, we searched for “solitary fibrous tumor”/exp OR “solitary fibrous tumor” AND thyroid without filters. The last search was performed in November 2025. The inclusion criteria comprised articles describing cases of confirmed diagnosis of SFTT, while articles describing unrelated neoplasms or articles that were not written in English were excluded, as reported in the PRISMA flow diagram (Figure 1). Two authors independently screened and selected the articles. Initially, they evaluated each study based on its abstract and title, identifying potentially eligible articles for further assessment. Full-length articles were then retrieved and reviewed in detail by the same two authors, according to predefined inclusion criteria. Subsequently, the authors independently extracted data from the articles that met these criteria. A standardized form was used to extract the following information: age, sex, tumor diameter, type of surgery performed, histological and immunohistochemical features, follow-up time and outcome. The quality of the case reports was evaluated using the JBI Critical Appraisal Checklist for Case Reports, a tool developed by the Joanna Briggs Institute to critically assess the methodological quality of case reports. The checklist includes eight items that evaluate how thoroughly and methodically the clinical case is described for a qualitative assessment of bias risk. For each item, the possible answers are yes, no, unclear, or not applicable. Although it does not officially assign a numerical score, following the approach used in other studies, we converted the responses into a quantitative score to enable comparison, assigning one point for each “yes” answer. The total score is calculated based on the number of “yes” responses, relative to the number of assessable items [10]. The aggregation of the data was carried out by evaluating the categorical variables of interest. These were expressed as percentage values, while continuous variables were expressed as the median and a minimum–maximum range, as appropriate. This systematic review has been registered in OSF (registration DOI: 10.17605/OSF.IO/AJEP8) [11].

## 3. Case Report

**Figure 2:**
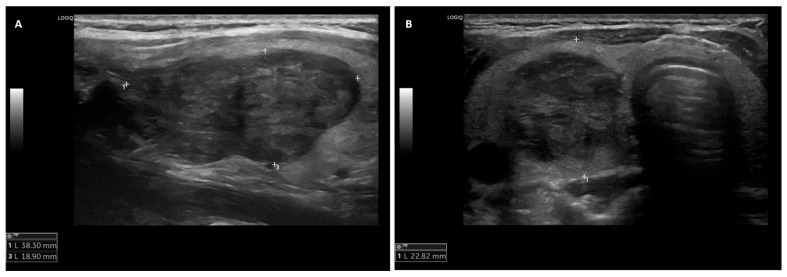
Ultrasound imaging of the thyroid right lobe. (**A**) Nodule longitudinal scan and (**B**) nodule axial scan.

**Figure 3:**
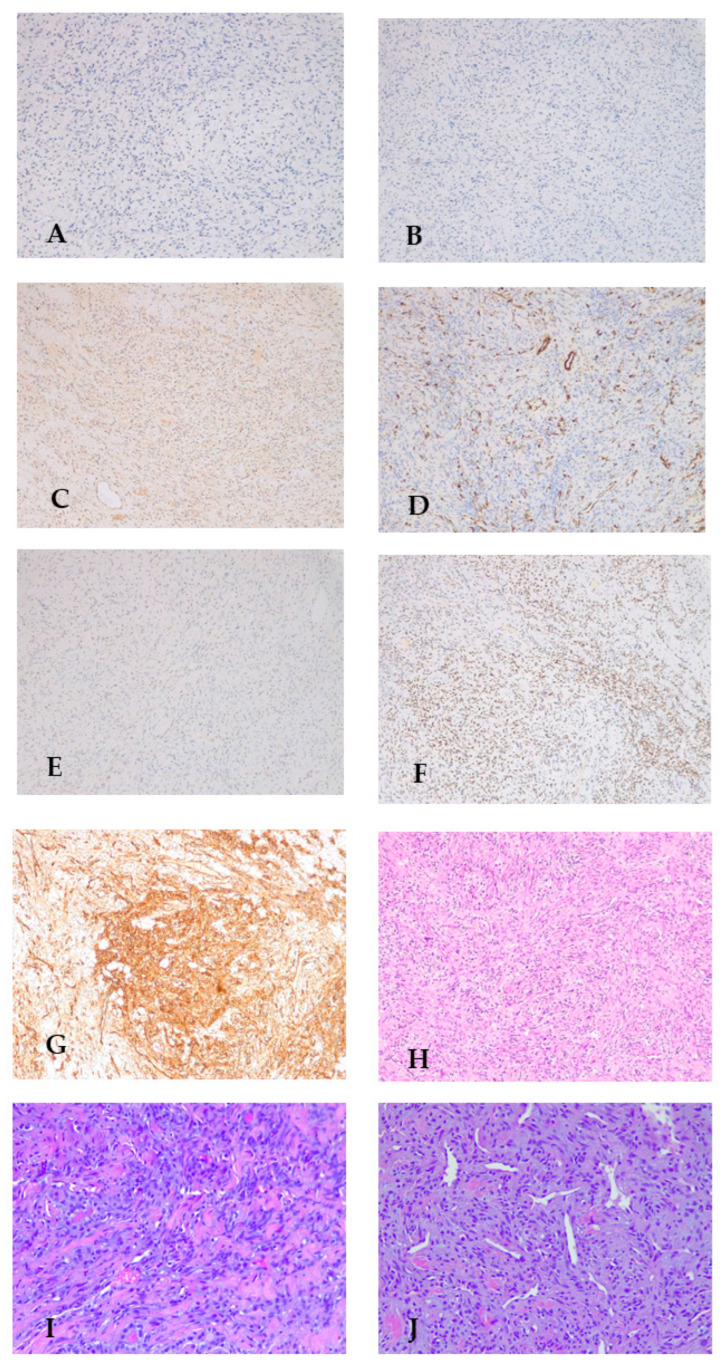
Microphotographs of the SFTT: (**A**) negative immunohistochemistry for S100 ×10; (**B**) negative immunohistochemistry for CK AE1 AE3 ×10; (**C**) negative immunohistochemistry for calcitonin ×10; (**D**) negative immunohistochemistry for ACT ML ×10; (**E**) negative immunohistochemistry for TTF1 ×10; (**F**) positive immunohistochemistry for STAT6 ×10; (**G**) positive immunohistochemistry for CD34 indicating ×10; (**H**) H&E coloring; (**I**) H&E coloring with “Staghorn pattern” ×20; and (**J**) H&E coloring with “patternless pattern” ×20.

A 36-year-old female with a history of smoking and previous breast cancer (treated with quadrantectomy, radiation therapy, chemotherapy, and subsequent therapy with LHRH analogs from 2017 to 2019) presented a palpable neck mass during a routine oncological follow-up in 2020. She was referred to Oncological Endocrinology at the IRCCS Regina Elena National Cancer Institute for thyroid nodules. A clinical examination and thyroid ultrasound revealed multiple thyroid nodules, including a hypoechoic nodule of 26 mm in diameter in the right lobe (Figure 2). The thyroid function tests were normal (TSH 1.3 mUI/L), and the calcitonin levels were within a normal reference range (<2 pg/mL). The patient denied previous cervical radiation therapy and any symptoms of dysphagia, dysphonia or dyspnea. She had no family history of thyroid disease or thyroid cancer. In August 2020, a fine needle aspiration biopsy (FNAB) of the 26 mm thyroid nodule in the right lobe was performed with a cytological benign diagnosis (TIR 2) [12]. In March 2022, given that the right thyroid nodule had grown to 38 mm in diameter, the FNAB was repeated, confirming the diagnosis of a TIR 2 nodule. In July 2023, an ultrasound showed that the right thyroid nodule was stable in size and echo structure compared to previous ultrasounds (38.3 mm). The thyroid ultrasound also revealed a darker-than-normal (hypoechoic) nodule measuring 13 mm in diameter in the right lobe. After FNAB of the latter nodule, the specimen of FNAB showed a marked cytological atypia, low colloid content, and histiocytes, which was consistent with the cytological diagnosis of TIR 3b classification (an indeterminate follicular lesion at a high risk of malignancy, with an estimated probability of cancer between 15% and 30%) [12]. When discussing the surgical options with the patient (lobectomy vs. total thyroidectomy), the patient preferred a total thyroidectomy, which was performed in October 2023. The histopathological examination revealed a 22 mm nodule in the right lobe of the thyroid, with positive staining for CD34 and STAT6, and negative for cytokeratins MNF116, cytokeratins AE1/AE3, EMA, Claudina, S100, Sox10, P40, P63, smooth muscle actin, CD56, calcitonin, chromogranin A, TTF1 and tireoglobulin, thus confirming the diagnosis of SFTT. The solitary fibrous thyroid tumor diagnosed at histology was a nodule in the right thyroid which, at the ultrasound, had a diameter of 38 mm and was found to be cytologically benign. The tumor was classified as low risk, based on the Demicco classification system, as well as on tumor size (22 mm), age (38 years old) with >4 mitoses per 10 high-power fields (HPF) and <10% necrosis [12,13] (Figure 3). Next Generation Sequencing (NGS) using the Archer FusionPlex Sarcoma panel showed that the NAB2-STAT6 fusion was consistent with the diagnosis of SFTT. Post-thyroidectomy neck ultrasounds performed in July 2024 and in May 2025 showed no evidence of lymph node involvement or loco-regional recurrence. The patient has been on levothyroxine replacement therapy since then and is currently in a state of euthyroidism. The patient is still under regular surveillance and is doing well after 26 months of clinical and instrumental follow-ups, undergoing neck and abdomen ultrasounds and chest x-rays.

## 4. Discussion and Systematic Review

Solitary fibrous tumors of the thyroid are rare neoplasms that are associated with nonspecific clinical presentation, which often overlap with other more common thyroid lesions. These tumors pose diagnostic challenges, which are mainly due to the lack of distinctive clinical and radiological features. In particular, cytological evaluations are problematic, as cytological findings are often non-diagnostic, benign, or can mimic thyroid cancer. For this reason, the diagnosis of SFTT is often difficult to diagnose preoperatively, therefore necessitating a definitive diagnosis, which heavily relies on postoperative histopathological and immunohistochemical evaluation. As of 2025, a total of 61 cases of SFTT have been documented in the literature and are summarized in Table 1. Most of the SFTTs had indolent benign behavior and presented rapidly growing, palpable neck masses that were rarely accompanied by symptoms such as dysphonia, dysphagia, and hemoptysis, while thyroid function was still typically within the normal reference range [3]. Thirty-two out of the 61 cases reported in the literature (52.45%) were female and 29 (47.54%) were male. The median age at diagnosis was 59 (28–88 years). The diagnosis of SFTT relies on a combination of imaging, cytology, histopathology, and molecular testing. Ultrasound imaging (US) is the primary imaging modality for thyroid nodules. SFTTs typically appear as hypoechoic, well-circumscribed nodules in USs. In thyroid ultrasounds, most SFTTs were described as hypoechoic nodules with some areas of cystic degeneration [1,3,7,8,14,15,16,17,18,19,20,21,22]. However, imaging alone cannot definitively diagnose SFTT, as it may mimic other thyroid neoplasms and other thyroid diseases [22]. In some cases, given the size of the thyroid nodule and in addition to studying the relationships with surrounding structures such as the trachea and esophagus, a computed tomography (CT) was performed, from which the SFTT transpired to be a solid formation, heterogeneous mass with low density, and well demarcated [3,5,7,14,16,17,20,21,23,24,25,26,27,28,29]. In a few cases, magnetic resonance imaging (MRI) was carried out, highlighting the SFTT as a heterogeneous formation with irregular margins [14,18]. Fine-needle aspiration biopsy is the gold standard procedure for the diagnosis of thyroid nodules. The SIAPEC 2014 classification system categorizes thyroid nodules from TIR2 (benign) to TIR5 (high malignancy risk). However, cytological findings in SFTTs are often non-diagnostic, due to their poor cellularity [3,7,15,16,25,26,30,31,32]. In some cases, they are described as benign [5,6,33], indeterminate [1,29,34] or neoplastic [1,18,24,35], requiring histological and immuno-histochemistry confirmation. In 16 cases, preoperative FNAB was not performed. In these cases, the patients underwent surgery due to the size of the nodule. The first-line treatment is surgery. In most of the reported cases (approximately 29.5%), patients underwent thyroid lobectomy; in 13 cases (21.3%), total thyroidectomy; in 14, subtotal thyroidectomy (23%); while in 15 cases (24.6%) the surgical treatment was not specified and a patient was not operated on. Approximately half of the cases were between 5 and 10 cm in size, while 44.3% (27 cases) were less than 5 cm and in no case did the tumor exceed 15 cm in diameter. Multi-focality has never been observed, but it is important to note that three cases reported an association with papillary thyroid carcinoma (PTC) in the opposite thyroid lobe [15,28,36]. Histologically, SFTTs were characterized by alternating hypocellular and hypercellular areas, with spindle cells arranged in a “patternless pattern” and abundant collagen deposition. Immunohistochemistry (IHC) is crucial for diagnosis, with CD34 and STAT6 marker positivity being highly specific for SFTT [4,13,37,38]. The NAB2-STAT6 fusion gene is a key molecular feature of SFTs. This fusion results from the combination of the NGFI-A-binding protein 2 (NAB2) gene with the signal transducer and activator of transcription 6 (STAT6) gene on chromosome 12q13. The most common fusion variant of intrathoracic SFTs is NAB2ex4-STAT6ex2, followed by NAB2ex6-STAT6ex16/ex17. In these cases, these variants are associated with younger patients and tend to exhibit more aggressive tumor behavior [39,40]. However, to date, there has been no established correlation between STAT6 fusion variants and prognosis in SFTTs, since there is no molecular study that allows us to observe the most common fusion found in the thyroid, as well as its prognostic impact. In our patient, a NAB2ex6-STAT6ex17 fusion was identified for the first time in the literature for SFTT. Despite the fact that the clinical course for our patient was indolent despite the fusion, it is not possible to draw conclusions based only on one case. NGS has become an invaluable tool for detecting these fusions, particularly RNA-Seq, which can show both known and novel fusion events. In patients reported in this review, the evaluation of the STAT6 fusion has primarily been conducted through immunohistochemistry, with only one reported case [41] utilizing next-generation sequencing (NGS), even though the type of fusion found in that instance is unknown [40,42]. While surgery remains the gold standard treatment, molecular profiling using NGS allows us to identify specific fusion variants. As has been done for other SFT sites, this molecular profiling allowed us to attribute a prognostic role to a specific fusion, defined as a risk factor associated with a greater or lesser degree of aggressiveness. Despite their benign nature, SFTTs carry a risk of local recurrence and metastasis, particularly in cases with high mitotic activity or necrosis, as observed in three cases in the literature [43,44,45]. The Demicco risk stratification system is a widely used, validated model to predict metastatic risk for SFTs. It categorizes tumors into low-, intermediate-, or high-risk groups, based on patient age, tumor size, and mitotic frequency, with a modified version (mDemicco) incorporating necrosis [13]. In the articles reported in the literature, it was not possible to identify the risk classes, due to the lack of data. In three cases, recurrence was detected, and distant metastases were observed, particularly in the lung [1,26] after two months [1] and five months [26] from surgery; in the liver [1,35]; and in the skin after four months [35] from surgery. The liver metastasis was treated with thermoablation and the lung nodule was kept under observation in the follow-up [1]. There was no information available regarding the therapeutic treatment adopted for the other metastases [26]. After surgery, patients were treated with levothyroxine in case of post-surgical hypothyroidism. Radioactive iodine therapy and TSH suppression were not found to be effective for SFTTs, as these tumors originate from mesenchymal (fibroblastic) cells rather than follicular epithelial cells, and therefore do not take up iodine or respond to TSH modulation. There are no guidelines on the treatment of solitary fibrous thyroid tumors. Since SFTT is a type of SFT, which is classified under soft-tissue sarcomas, any disease progression should refer to the treatment guidelines for soft-tissue sarcomas. The SEOM Clinical Guideline for the management of soft tissue sarcoma [46] in the case of metastatic or locally advanced malignant SFT recommends the use of pazopanib, an inhibitor of protein kinases receptors expressed on the cell surface involved in tumor growth and proliferation, such as VEGFR, PDGFR and KIT. Currently, there is no standard follow-up for solitary fibrous tumors of the thyroid, but given their indolent course in the vast majority of cases, we suggest that it could be assimilated to the NIFTP (non-invasive follicular thyroid neoplasia with papillary-like nuclear features) for their shared natural history, despite being biologically distinct entities, as SFTT is a mesenchymal tumor, while NIFTP is an epithelial neoplasm [47]. Specifically, both entities generally show indolent clinical behavior and a low risk of recurrence, which may support a conservative follow-up approach. This study has some limitations, primarily because it examines clinical cases of a rare type of tumor for which there are currently no established follow-up guidelines. Furthermore, many cases do not report the main histological features such as necrosis and mitosis, which are necessary for the Demicco risk stratification model. In the future, it is expected that all clinical cases will report these types of data in order to allow for better prognostic evaluation and comparison between future case studies. In nearly all cases reported in the literature, the STAT6 gene fusion was detected by using immunohistochemistry, a method that is relatively easy to perform. In future studies, it would be worthwhile to identify STAT6 fusions using NGS, to try to identify variants with prognostic value, with the goal of combining these findings—similar to the approach taken for intrathoracic SFTs—to better identify the prevalence of fusion variants expressed in SFTTs and those with a worse prognosis.

## 5. Conclusions

SFTTs are rare and difficult-to-diagnose tumors that require a multidisciplinary approach for accurate diagnosis and management. The combination of imaging, cytology, histopathology, and molecular testing is essential to distinguish SFTTs from other thyroid malignancies. In these cases, although cytology is the gold standard for diagnosing thyroid nodules, its efficacy in diagnosing SFTTs is limited; therefore, the combination of histology and immunohistochemistry is essential. The NAB2-STAT6 fusion is a key molecular feature that not only aids in diagnosis but also offers potential prognostic value, and, like in other tumors, therapeutic targets for future treatments. Advances in molecular diagnostics, particularly NGS, have improved diagnostic accuracy and provided insight into the genetic basis of SFTTs. The Demicco classification system offers important prognostic information that aids in clinical management and follow-up strategies. Although surgical excision remains the mainstay of treatment, long-term surveillance is essential to monitor for recurrence or metastases. Further research and case studies are needed to further clarify the behavior and molecular characteristics of SFTTs, particularly in relation to their malignant potential.

## Figures and Tables

**Table 1:** Summary of reported cases of solitary fibrous tumor of thyroid.

No.	Ref.	Age	Sex	Site	Size (cm)	Tx	CD-34	STAT6	Necrosis	Mitosis ^*^	Outcome	FU (Months)	Quality Sec JBI
1	[19]	44	F	R	6.5	TL	NA	NA	NA	Rare	NED	60	
2		61	M	L	6	ST	NA	NA	NA	-	NED	48	H
3		32	F	R	3.5	TL	NA	NA	NA	Rare	NED	60	
4	[30]	51	M	R	5	TT	NA	NA	-	-	NA	NA	I
5	[48]	48	F	R	8	ST	+	NA	-	-	NED	NA	H
6	[49]	43	F	L	4	ST	NA	NA	-	>4/10	NED	160	H
7	[50]	28	F	NA	2.5	NA	NA	NA	NA	>4/10	NA	lost	H
8	[31]	43	F	L	3.5	NA	+	NA	-	2/10	NA	lost	
9		52	M	L	2.5	NA	+	NA	-	-	NA	lost	
10		44	M	L	2	NA	+	NA	-	1/10	NA	lost	H
11		64	F	R	4.5	NA	+	NA	-	2/10	NED	60	
12		53	M	L	6	NA	+	NA	-	1/10	NED	60	
13		47	F	R	4.5	NA	+	NA	-	-	NED	48	
14		64	F	L	3	NA	+	NA	-	-	NA	NA	
15	[14]	56	M	R	8	TL	+	NA	-	-	NED	12	I
16	[51]	36	M	L	6	TT	+	NA	-	-	NED	25	H
17	[23]	68	M	L	9.7	ST	+	NA	-	-	NED	54	H
18	[24]	61	M	L	5	ST	+	NA	-	-	NA	lost	H
19	[25]	64	M	R	5	ST	+	NA	-	-	NED	60	H
20		41	M	R	3	TL	+	NA	-	-	NED	48	
21	[15]	70	F	R	1.5	TT	+	NA	-	-	NED	6	H
22	[52]	45	M	R	5	TL	NA	NA	NA	NA	NA	NA	H
23	[16]	61	M	R	3.5	TT	+	NA	-	-	NED	60	H
24		42	F	R	4.7	ST	+	NA	-	-	NED	84	
25	[53]	51	M	L	7	NA	+	NA	NA	NA	NA	NA	H
26	[54]	69	F	NA	2.2	NA	+	NA	NA	NA	NA	NA	H
27	[26]	76	F	R	4	ST	+	NA	+	High	AWD	6	H
28	[17]	58	M	L	8	TL	+	NA	-	<1/10	NED	NA	H
29	[8]	37	M		4	ST				<1/10	NED	12	H
30	[7]	47	F	L	5.2	TL	+	NA	-	NA	NED	9	H
31		59	M	R	7	TL	+	NA	+	<1/10	NED	31	
32	[27]	88	F	IT	9	ST	+	NA	+			36	H
33	[18]	68	M	L	8	NA	+	NA	NA	NA	NED	9	H
34	[6]	60	F	R	13.8	TL	+	NA	-	6/10	NA	NA	H
35	[55]	61	F	L	10.5	NA	NA	NA	NA	NA	NED	19	H
36	[56]	74	M	R	12	TT	+	NA	-	Rare	NED	24	H
37	[28]	78	M	R	3.5	ST	+	NA	-	-	NED	12	H
38	[3]	41	F	L	11	TT	+	+	-	4/10	NED	10	H
39	[32]	44	F	NA	7	TL	+	+	-	Rare	NED	41	H
40		45	F	NA	8.2	TL	+	+	-	Rare	NED	28	
41		52	M	NA	7	TL	+	+	-	Rare	NED	5	
42	[5]	59	M	L	5.5	TL	+	+	-	-	NED	17	H
43	[33]	34	F	L	5.1	TT	+	NA	-	-	NED	NA	H
44	[1]	66	M	L	6	TT	+	+	-	6/10	AWD	17	
45		45	F	L	4.5	TT	+	+	-	<1/10	NED	12	H
46		61	F	L	2.8	TT	+	+	-	1–2/10	NED	10	
47	[34]	82	M	NA	5	None	-	+	NA	NA	AWD	132	H
48		60	F	NA	1.6	TL	NA	+	NA	NA	NED	108	
49	[41]	65	F	NA	1	NA	+	-	NA	Rare	NA	NA	
50		72	M	NA	4.3	NA	-	+	NA	Rare	AWD	12	H
51		80	M	NA	5	NA	+	+	NA	Rare	NA	NA	
52	[35]	46	M	R	4	TL	+	+	-	>4/10	AWD	4	H
53	[20]	63	M	L	6	ST	+	+	-	-	NED	18	H
54	[57]	45	F	L	5	TL	+	+	-	-	NED	60	H
55	[58]	61	F	R	4.3	TT	+	NA	+	>4/10	NED	ongoing	H
56	[21]	73	F	R	13.8	ST	+	+	-	NA	NED	14	H
57	[29]	70	F	L	13	TL	+	+	NA	-	NA	NA	H
58	[59]	72	F	L	2	TT	+	+	NA	NA	NA	NA	H
59	[36]	46	F	R	5.5	TT	+	+	-	-	NED	9	H
60	[60]	62	F	R	4	ST	+	+	-	3/10	NA	NA	H
61	[61]	63	M	R	4.9	ST	+	NA	NA	NA	NED	60	H

Ref.: reference; Tx: treatment; FU: follow-up; M: male; F: female; R: right; L: left; NA: not available; TL: thyroid lobectomy; ST: subtotal thyroidectomy; TT: total thyroidectomy; NED: no evidence of disease; and AWD: alive with disease (recurrence and distant metastases). * Number of mitoses on 10 high-power fields (HPF); JBI: Joanna Briggs Institute; H: high quality; and I: intermediate quality.

## Data Availability

Data analyzed in this review are included in this article. Further inquiries can be directed to the corresponding author.

## Abbreviations

The following abbreviations are used in this manuscript:

SFTTs	Solitary fibrous tumors of the thyroid
SFTs	Solitary fibrous tumors
CNS	Central nervous system
FNAB	Fine needle aspiration biopsy
HPF	High-power fields
NGS	Next-generation sequencing
PTC	Papillary thyroid carcinoma
US	Ultrasound
IHC	Immunohistochemistry
NIFTP	Non-invasive follicular thyroid neoplasia with papillary-like nuclear features
TT	Total thyroidectomy
TL	Thyroid lobectomy
NED	No evidence of disease
AWD	Alive with disease (recurrence and distant metastases)
NA	Not available
ST	Subtotal thyroidectomy
M	Male
F	Female
R	Right
L	Left
ACT ML	Smooth muscle actin
H&E	Hematoxylin and eosin
WHO	World Health Organization
PRISMA	Preferred Reporting Items for Systematic Reviews and Meta-Analyses
EMBASE	Excerpta Medica database
SIAPEC	Società Italiana di Anatomia patologica e di Citopatologia Diagnostica
NAB2-STAT6 gene fusion	NGFI-A-binding protein–signal transducer and activator of transcription 6 gene fusion
VEGFR	Vascular–Endothelial Growth Factor
PDGFR	Platelet-Derived Growth Factor Receptor
CT	Computed Tomography
MRI	Magnetic Resonance Imaging
Tx	Treatment
FU	Follow-up
JBI	Joanna Briggs Institute
H	High
I	Intermediate
